# Local and regional rarity in a diverse tropical fish assemblage

**DOI:** 10.1098/rspb.2012.2076

**Published:** 2013-01-22

**Authors:** A. P. Hercos, M. Sobansky, H. L. Queiroz, A. E. Magurran

**Affiliations:** 1 Mamirauá Institute for Sustainable Development (MISD), Estrada do Bexiga 2584, CEP 69470-000, Tefé, Amazonas, Brazil; 2 INPA/BADPI, Department of Aquatic Biology and Fisheries, National Institute for Amazonian Research, Avenida André Araújo, 2936, CEP 69060-001, Manaus, Amazonas, Brazil; 3 School of Biology, Centre for Biological Diversity and Scottish Oceans Institute, University of St Andrews, St Andrews, Fife KY16 8LB, UK

**Keywords:** species abundance distribution, occurrence, várzea, freshwater fish, rare biosphere, Amazon

## Abstract

Because most species in an ecological assemblage are rare, much of the species richness we value is due to taxa with few individuals or a restricted distribution. It has been apparent since the time of ecological pioneers such as Bates and Darwin that tropical systems have disproportionately large numbers of rare species, yet the distribution and abundance patterns of these species remain largely unknown. Here, we examine the diversity of freshwater fish in a series of lakes in the Amazonian várzea, and relate relative abundance, both as numbers of individuals and as biomass, to the occurrence of species in space and time. We find a bimodal relationship of occurrence that distinguishes temporally and spatially persistent species from those that are infrequent in both space and time. Logistic regression reveals that information on occurrence helps distinguish those species that are rare in this locality but abundant elsewhere, from those that are rare throughout the region. These results form a link between different approaches used to evaluate commonness and rarity. In doing so, they provide a tool for identifying species of high conservation priority in poorly documented but species rich localities.

## 1. Introduction

In 1863, Bates [1] noted that while the density of butterflies in the Amazon was similar to that in UK, the number of species represented by those individuals was vastly greater. Growing concern about the state of the world's biological diversity, much of which is found in tropical regions, underlines the need to learn more about the structure of these diverse assemblages, the majority of which are poorly documented [2,3]. However, we do know that much of this unknown diversity will be comprised of rare taxa. The universal pattern in species abundance distributions is that both rare and common species are found in every community [4], with the fraction of rare species increasing in rich habitats. Indeed, surveys of tropical arthropod assemblages routinely find that many species are present in a sample only as singletons or represented by a few individuals [5,6]. This preponderance of rare species raises particular problems from a conservation standpoint. Because rarity is thought to increase the risk of extinction, either through demographic stochasticity or because species that occupy a restricted habitat are vulnerable if it is modified, being rare is a factor than can be taken into account in decisions to list threatened species [7] or in developing local management plans. On the other hand, if most species are rare it is difficult to know which ones to prioritize. Moreover, while average local abundance tends to be higher for species that occur in many sites [8–10], species that are rare in one locality are not necessarily scarce everywhere because their abundance may be depressed as a result of local conditions (e.g. unsuitable habitat) [11] or through random chance. An important challenge, therefore, for researchers and conservationists working in species rich but poorly documented localities, is to distinguish species that are rare throughout their range from those that just happen to be rare in a particular site.

To address this issue, we need to learn more about the structure of tropical assemblages—particularly those that have been relatively little studied. While a comprehensive evaluation of tropical biodiversity would be an immense and impracticable task [12,13], we do know that different types of diversity pattern(s) are linked [14–17]. McGill [16], for instance, points out that extent and abundance tend to covary, meaning that there will typically be some species that are common in terms of range size, occupancy and abundance, and others that are rare by the same metrics. He dubs this the ‘common is common’ pattern. By the same token, information on different aspects of rarity could help distinguish species that have low abundance in the region as a whole—the ‘rare throughout’ species—from those that happen to be rare in a particular locality or habitat—the ‘rare locally’ species.

Here, we examine the diversity of fish in lakes within the Amazonian flooded forest (várzea) to ask how the pattern of species occurrence, in space and in time, is linked to the relative abundance of species. Occurrence is a measure of the number of sites or samples that a species is present in [17]. A bimodal or U-shaped relationship of species occurrence is often seen if the quadrats used to sample an assemblage are divided into bins, representing species that are progressively more widespread [16,18]. Interestingly, a similar bimodal pattern of species occurrence appears if data are accumulated at the same place through time [19]. Given the extent to which autocorrelation influences spatial patterns of biodiversity [16], it seems likely that species that occur frequently in space are the same as those that are persistent through time. We therefore predict that if we track species simultaneously over space and time, we will detect a mode of species infrequent in both space and time, and a second mode of species that are prevalent in both space and time. This pattern will be strongest at local scales, where behaviour and ecology promote clumping of individuals. Species abundance distributions on the other hand tend to be unimodal (but see [20])—often resembling a lognormal. However, Preston [21] asserted that species abundance and species occurrence distributions are different expressions of the same underlying pattern. We therefore argue that species abundance distributions can be deconstructed to reveal ordered groupings of species that are increasingly persistent in space or time. Finally, we propose that species that occur in the ‘persistent’ mode, yet have low numerical abundance, are more likely to be in the ‘rare locally’ than in the ‘rare throughout’ category. We suggest that these ‘signatures’ of rarity can be a useful aid to researchers characterizing the diversity of rich assemblages and to managers charged with conserving these systems.

**Figure 1.**
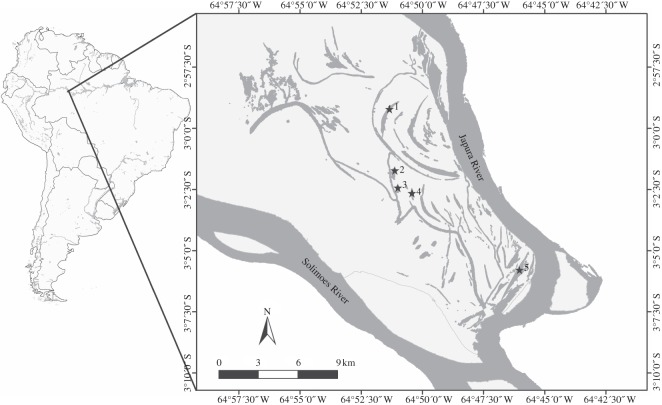
Study area (with sampled lakes numbered 1–5).

We test these ideas using data collected during an intensive survey of fish communities in five small lakes (figure 1) sampled monthly for 18 months. This highly seasonal habitat is inundated for around 6 months per year [22]. Very few of the fish species found here have had their conservation status formally evaluated (see the electronic supplementary material, S1).

## 2. Material and methods

### (a) Site description

This study was carried out at the Mamirauá Sustainable Development Reserve (MSDR), in the Brazilian Central Amazon floodplain (figure 1), a wetland site of international importance recognized under the UN Ramsar Convention [23]. Mamirauá Reserve is the largest protected area of flooded forests—known as *várzea*—in Brazil. The reserve's 1 124 000 ha are completely flooded for 3 to 6 months every year during the flooding pulse. During this time, the water level varies by more than 10 m [22]. Four seasons can be identified, based on water level monitoring since 1990 [17]. These are (i) the *cheia*, or high water season, from May until mid-July; (ii) the *vazante* or falling water season from mid-July to September; (iii) the *seca* or low water season in the months of September, October and November; and (iv) and the *enchente* or rising water season from December to April.

The mosaic of forests [24] and water bodies, lakes and channels, at Mamirauá is typical of the várzea environment (figure 1). These water bodies support a large fish fauna [25], with diverse communities in each of the major aquatic habitats present [26].

Floating meadows form an important aquatic habitat at Mamirauá Reserve [26,27] and vary in extent as the water level rises and falls [28]. Although these meadows can be highly diverse, they are usually dominated by a small number of plant species. The more abundant grass and macrophyte species found at Mamirauá are the Poaceae *Paspalum repens* (among other *Paspalum* species), *Echinochloa polystachia*, the Potenderiaceae *Eichornia crassipes*, the Araceae *Pistia stratiotes*, the Azollaceae *Azolla* spp. and the Salviniaceae *Salvinia minima* [26].

### (b) Sampling

Five lakes in Mamirauá Reserve (lakes Araçazinho, Juruá Grande, Juruazinho, Pagão and Tracajá; figure 1) were sampled monthly in the period from September 2003 to March 2005. The lakes are inundated during the high water season, but during the dry season, it is possible to calculate lake size; this varied from 11.1 to 102.3 ha. Each sample was made up of five sample units. These units consisted of 16 m^2^ of vegetation which was separated from the larger blocks of floating meadows, surrounded by a seine net (multifilament; 2 mm mesh size, 30 m long and 6 m wide), and then lifted into a boat. All plants were removed from the net and returned to the sample site. All fish were then collected and transported to the field laboratory in buckets where they were identified, measured and weighted. Whenever possible, fish were returned alive to the local water bodies, but when identification was not possible in the field, they were humanely killed and taken to the laboratory at Tefé, the nearest town in Amazonas State. In such cases, fish specimens were preserved in a 10 per cent formalin solution for transportation, and transferred to a 70 per cent alcohol solution in the laboratory. Total effort, which was consistent throughout, was 400 m^2^ of floating meadow per month across all five lakes. Fish were identified using the literature including [29–32] and the fish collection at Mamirauá Institute.

### (c) Data analysis

**Figure 2.**
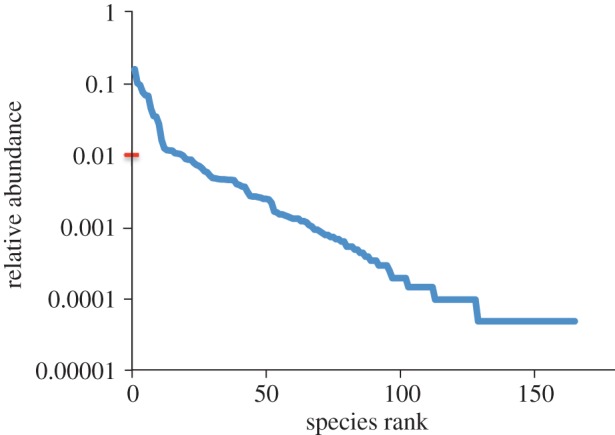
Rank abundance plot (relative abundance of species in relation to species rank) of the várzea lake fish assemblage. Species with more than 1% of total abundance (0.01—indicated by the dash on the *y*-axis) are designated common species.

In line with [33], we defined species as rare if they had less than 1 per cent of the total number of individuals overall. This gave 20 common species and 145 rare species and coincided with an inflection point in the rank abundance plot of the assemblage (figure 2). Rare species were then assigned to the categories ‘rare locally’ (i.e. those rare in these samples and thus in the floating meadow habitat in lakes, but frequently encountered elsewhere in the reserve) and ‘rare throughout’ (i.e. rare in the other aquatic habitats present in the reserve). To do this, we assembled data that had been collected during extensive routine surveys in six other habitat types within the reserve. These habitats were: open water; banks and margins; flooded forest; floating meadow within channels; forest temporary pools; and submerged dead trees. We were able to extract information on both the presence and relative abundance of 142 out of our 145 rare species in each of the other habitats (three species were excluded from the analysis owing to incomplete information). We used these independent data on the distribution and abundance of the species elsewhere in the reserve to distinguish between the two types of rare fish in our lake floating meadow fish. To explore how our conclusions are influenced by the way the distinction between ‘rare locally’ and ‘rare throughout’ is reached, we repeated this categorization four times using different criteria. The definitions of ‘rare locally’ adopted were (i) present in at least two other habitat types (*n* = 63 ‘rare locally’ species); (ii) present in at least three other habitat types (*n* = 41 ‘rare locally’ species); (iii) present in at least two other habitat types OR ≥ 0.5 per cent of total abundance when present anywhere (*n* = 74 ‘rare locally’ species); (iv) present in at least two other habitat types OR ≥ 1 per cent of total abundance when present anywhere (*n* = 69 ‘rare locally’ species). For more details, see electronic supplementary material, S2.

Logistic regression was used to ask whether information on persistence can be used to deduce a fish's rarity status, in other words to help distinguish between species that are ‘rare locally’ and those that are ‘rare throughout’. Here, the response variable was status (‘rare locally’ or ‘rare throughout’) while the predictor variables were body size (log_10_ mean wet weight in grams), and persistence (either the number of lakes (1–5) or the number of seasons (1–4) a species was detected in). Because researchers will typically have either spatial or temporal data rather than both, lake number and season number were analysed separately. Lake number and season were treated as factors. We repeated the analysis for each of our four definitions of ‘rare locally’ species. Analyses were conducted in R [34].

## 3. Results

**Figure 3.**
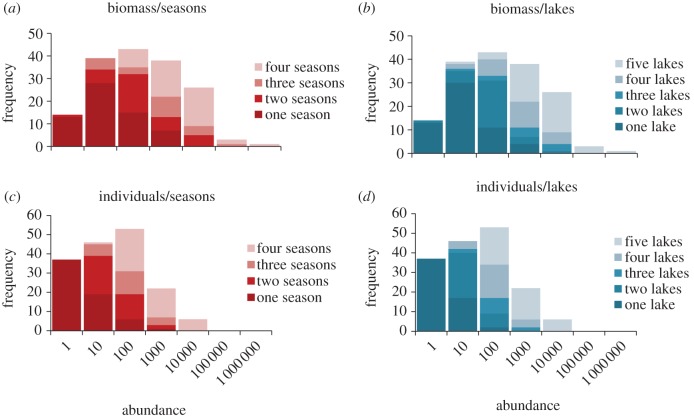
Species abundance distributions showing the frequency of species in log_10_ abundance classes for biomass (*a*,*b*) and numerical abundance (*c*,*d*). The legends indicate species that are present in one, two, three or four seasons, or one, two, three, four or five lakes.

Sampling yielded greater than 20 000 individual fish belonging to 165 species in 99 genera and in 29 families. The distribution of biomass (figure 3) follows a typical lognormal distribution. Dominant species (with total biomass greater than 10 000 g) include the cichlids *Cichla monochulus* (peacock cichlid), *Cichlasoma amazonarum* (Amazon cichlid), *Mesonauta insignis* (flag cichlid) and *Pterophyllum scalare* (angelfish)*.* The distribution of numerical abundance, on the other hand (figure 3), resembles a truncated lognormal with an excess of species in the low abundance classes. Thirty-seven species are represented only as singletons, whereas numerically abundant species (*n* > 1000) include both cichlids (*Mesonauta insignis*, *Cichlasoma amazonarum*) and characids (e.g. *Pygocentrus nattereri* (red-bellied piranha), *Mylossoma duriventre* (silver dollar)*, Moenkhausia intermedia* (scissortail tetra) and *Ctenobrycon spilurus* (silver tetra)). For a list of species, see electronic supplementary material, S1.

**Figure 4.**
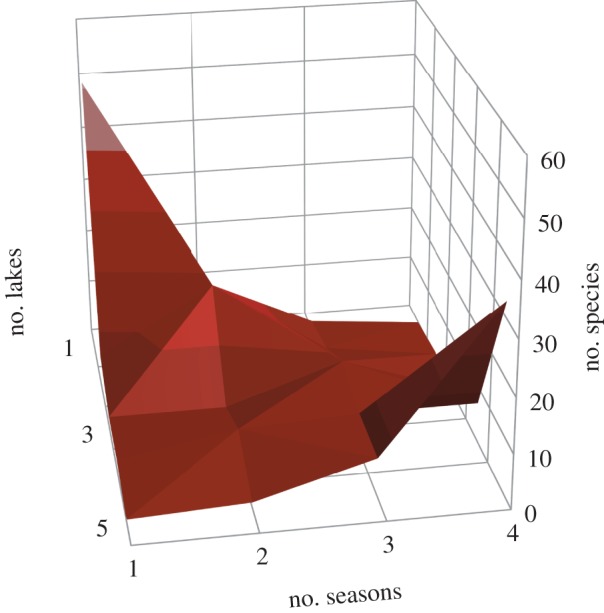
Frequency of species in relation to the number of lakes, and the number of seasons, in which they occur.

Figure 4 reveals two distinct modes of occurrence, one of species found in a single season and lake, the other of species present in most lakes and most seasons. When these occurrence patterns are superimposed on the overall species abundance distributions (figure 3), it is clear that for both abundance currencies there is an ordered sequence of occurrence with increasingly persistent species progressively dominating the larger abundance classes.

The logistic regression analysis indicates that the less frequently a rare species is detected in either space or time, the more likely it is to be ‘rare throughout’ the region. The analysis treated space (number of lakes) and time (number of seasons) as categorical values; their overall effect is shown in table 1. Table 1 also indicates that the different methods used to distinguish the ‘rare locally’ and ‘rare throughout’ species lead to similar conclusions indicating that local information on the occurrence of a species in either space or time is a robust indicator of its status. The details of the individual logistic regressions are provided in the electronic supplementary material, S3, which also graphs the predicted probabilities. From these graphs, it is clear that both the lake effect and season effect are stronger for species with larger body sizes. The distribution of body size is shown in the electronic supplementary material, S4. There is no interaction between body size and numbers of seasons or lakes. Overall, it appears that fish that are present in only one or two seasons, or fewer than four lakes, are the most likely to fall into the regionally rare group (see the electronic supplementary material, S3).

**Table 1.** Wald test of the overall effect of space and time when predicting the probability that a species is ‘rare throughout’ rather than ‘rare locally’. The predictor values of interest here are the number of lakes or the number of seasons a species was present in during the investigation of lake floating meadow habitat; lake number and season number are treated as factors (see text for more details). The analysis, which is repeated for each of the definitions of ‘rare locally’ (see electronic supplementary material, S2), uses the *aod* package in R [35]. Full details of the logistic regression analyses are provided in electronic supplementary material, S3.

definition of ‘rare locally’	space (lakes)	time (seasons)
two habitats; *n* = 63 ‘rare locally’ species	**χ**^2^ = 12.3, d.f. = 4, *p* = 0.015	**χ**^2^ = 13.7, d.f. = 3, *p* = 0.003
three habitats; *n* = 41 ‘rare locally’ species	**χ**^2^ = 8.8, d.f. = 4, *p* = 0.067	**χ**^2^ = 10.0, d.f. = 3, *p* = 0.018
two habitats or ≥0.5%; *n* = 74 ‘rare locally’ species	**χ**^2^ = 14.3, d.f. = 4, *p* = 0.007	**χ**^2^ = 11.9, d.f. = 3, *p* = 0.008
two habitats or ≥1%; *n* = 69 ‘rare locally’ species	**χ**^2^ = 14.8, d.f. = 4, *p* = 0.005	**χ**^2^ = 14.1, d.f. = 3, *p* = 0.003

## 4. Discussion

These results reveal, as expected, that many species of fish in this rich Amazonian system are rare, in terms of both numerical abundance and biomass. However, while the relative abundances of these species take the classic graded form from rare to common, resembling a typical ‘lognormal’ pattern (which is truncated in the case of numerical abundance), species occurrences fall into two clusters: a group of species that are infrequent in both space and time, and a group that is persistent in space and time. We can draw on this information to help distinguish between those species, which, while rare in terms of abundance in this habitat type and lake system, are common elsewhere, and those that are rare throughout. Because temporal and spatial occurrence are linked, information on either can be used to deduce species status.

This ability to pinpoint species that may be of particular conservation concern [7], and to discriminate between low abundance taxa is important, particularly in areas rich in biological diversity. Comprehensive surveys of diverse but little-studied systems require unrealistic inputs of time and resources [2]. Indeed, H.W. Bates's inventory of insects in the environs of Mamirauá, over 150 years ago, while far from complete, remains the best there is [12]. As we have shown, it is possible to make inferences about the status of species, and the likelihood that they are regionally rare, from small-scale surveys and using the types of data that field researchers typically gather. While this information could aid managers assessing species, particularly in the absence of more detailed data on range and local population size [36], the approach we have outlined here is potentially useful in other contexts too. For example, microbial communities are as yet very incompletely surveyed, and one of the biggest challenges facing researchers working in this area is characterizing the ‘rare biosphere’ [37]. Information on both occurrence (in space and/or time) and abundance could be used to distinguish transiently rare microbial taxa from those that are globally rare. Microbial systems, in the same way as the aquatic communities that inhabit the várzea, experience high levels of spatial and temporal turnover. When turnover is reduced, we would expect to see a lower ‘rare’ mode and a larger ‘common’ mode of occurrence, but that the overall patterns would be conserved [19]. Turnover will also influence the degree of spatial and temporal autocorrelation in a system [16].

Our findings have other implications. For example, the occurrence data reveal how a species abundance distribution is constructed by overlays of species that are increasingly persistent in time, and in space. Species abundance distributions are one of the oldest methods of examining diversity data, and provide a clear means of describing and comparing communities [4,38]. However, there is still considerable debate about why species abundance distributions take the form they do, and how they are shaped by the currency used to measure abundance [39]. Deconstructing species abundance distributions provides a means of examining the mechanisms that underpin them [4]. Our results show that species are ordered by occurrence with infrequent species predominant at the rare end of the distribution. Interestingly, we found that this pattern is the same for both numerical abundance, and biomass, and whether the distributions are assembled using temporal or spatial data. This knowledge will aid attempts to predict species abundances from occurrence data [40].

Although infrequent and persistent species are ordered in the same way in species abundance distributions that use the alternative currencies of numerical abundance and biomass, the form of these distributions differs. When biomass is plotted, the distribution is unimodal and close to a textbook representation of a lognormal distribution. By contrast, when abundance is measured as numbers of individuals, the distribution is asymmetric with an excess of species in the low abundance classes. This pattern is similar to the one that Connolly *et al.* [41] detected when assessing the diversity of marine fish and corals. Connolly and co-workers argued that it is only at very large scales (*ca* 10 000 km) that lognormal distributions of numerical abundance are fully unveiled. At the scale of the local community, differences in the shape of the species abundance distributions of biomass and numerical abundance can be explained by body size and the utilization of the spatial niche [42]. Resource allocations will primarily involve the persistent taxa—species that are usually present and generally more abundant. However, we suggest that the structural complexity of the floating meadow habitat [43,44] enables rare species to persist and this, combined with the cyclical inundation regime, contributes to high turnover that underpins the diversity of this system.

The observed separation of infrequent and persistent species resonates with Hanski's [45] core-satellite hypothesis. However, the core-satellite hypothesis predicts relatively rapid shifts in abundance so that a rare species may become a core one, and vice versa [46]. Our data imply that temporal and spatial rarity are linked, though the relatively short-term nature of the sampling does not provide a definitive test of this. Nonetheless, other investigations, where species have been tracked through decades, suggest that core species maintain their abundance and rank through time [47–49].

To date, most emphasis has been on spatial patterns of biological diversity. Growing concern about the fate of the world's biota underlines the need to understand and measure how ecological communities change through time [50]. Although temporal and spatial data can have different characteristics (such as the extent to which datasets are bounded) ‘space for time’ substitution is a tool used to interpret biodiversity patterns [51]. Our results illustrate how this substitution can work and underline the links between different patterns of diversity [16].
